# Synaptic activity and Alzheimer's disease: a critical update

**DOI:** 10.3389/fnins.2015.00423

**Published:** 2015-11-04

**Authors:** Davide Tampellini

**Affiliations:** U 1195 Institut National de la Santé et de la Recherche Médicale, Université Paris Sud, Université Paris-SaclayLe Kremlin-Bicêtre, France

**Keywords:** Alzheimer, synapses, synaptic activity, beta-amyloid, tau

## Abstract

Synapses have been known for many years to be the crucial target of pathology in different forms of *dementia*, in particular Alzheimer's disease (AD). Synapses and their appropriate activation or inhibition are fundamental for the proper brain function. Alterations in synaptic/neuronal activity and brain metabolism are considered among the earliest symptoms linked to the progression of AD, and lead to a central question in AD research: what is the role played by synaptic activity in AD pathogenesis? Intriguingly, in the last decade, important studies demonstrated that the state of activation of synapses affects the homeostasis of beta-amyloid (Aβ) and tau, both of which aggregate and accumulate during AD, and are involved in neuronal dysfunction. In this review we aim to summarize the up-to-date data linking synaptic/neuronal activity with Aβ and tau; moreover, we also intend to provide a critical overview on brain activity alterations in AD, and their role in the disease's pathophysiology.

## Relation between synaptic activity and Aβ homeostasis

Synapses are considered to be an early site of dysfunction/pathology in AD (Selkoe, 2002), and loss of synapses is the best pathologic correlate of cognitive impairment in AD patients (Terry et al., 1991). For many years it has been known that Aβ peptide, one of the main players in AD pathology derived by the β- and γ-secretase cleavage of the amyloid precursor protein (APP), can induce morphological and functional alterations to synapses and synaptic plasticity (Selkoe, 2002; Coleman and Yao, 2003; Almeida et al., 2005). Surprisingly, elegant studies from Roberto Malinow's and David Holtzman's groups demonstrated that, in turn, synaptic activity affects Aβ: increased activity enhances secretion of Aβ, while reduced activity inhibits it (Kamenetz et al., 2003; Cirrito et al., 2005). This discovery represented an important breakthrough in the field. For the first time it was shown that Aβ homeostasis was controlled by the main target of Aβ itself: synapses and their state of activation.

Since inhibition of synaptic activity reduces Aβ secretion, reduced activity appears to be positive for AD. Thus, our and other groups investigated the effect of chronic inhibition of synaptic activity on transgenic AD mice to explore whether it could indeed protect synapses. Chronic reduction of synaptic activity by unilateral whisker ablation, diminishes plaque burden in the deafferented barrel cortex of AD mice (Tampellini et al., 2010; Bero et al., 2011). On the contrary, chronic unilateral activation of the perforant pathway by optogenetic light activation (an experimental approach which also induced epileptic seizure in the studied AD mouse cohort), increases the amount of amyloid plaques (Yamamoto et al., 2015). Despite the reduction of amyloid plaques, we found that levels of synaptophysin and number of synapses were also reduced by chronic inhibition of activity in AD, but not wild-type, mouse brains (Tampellini et al., 2010). In addition, reduced activity worsened memory impairments in AD mice compared to controls (Tampellini et al., 2010). Intriguingly, in brain areas where plaques were reduced intraneuronal Aβ was increased, and presented an inverse correlation with levels of synaptophysin (Tampellini et al., 2010). Intraneuronal Aβ accumulation has been observed in human AD brains, in several rodent models of AD (Gouras et al., 2000; D'Andrea et al., 2001; Busciglio et al., 2002; Mori et al., 2002; Oddo et al., 2003; Cataldo et al., 2004; Echeverria et al., 2004; Cruz et al., 2006; Oakley et al., 2006; LaFerla et al., 2007), and, more recently, also in brains of aged mouse lemur primates (Roy et al., 2014). Within neurons, Aβ accumulates on the outer membrane of multivesicular bodies, both in somas and neurites (Takahashi et al., 2002; Casas et al., 2004; Cataldo et al., 2004), and is associated with early pathological alterations in dendrites, axonal terminals and synapses (Takahashi et al., 2004; Bayer and Wirths, 2010; Gouras et al., 2010). Clearance of intraneuronal Aβ by immunotherapy was shown to protect synapses and improve memory in *in vitro* and *in vivo* models of AD (Billings et al., 2005; Tampellini et al., 2007).

On the other hand, synaptic activity induced by specific activation of synaptic (but not extra synaptic) NMDA receptors (Lu et al., 2001), produced beneficial effects on AD transgenic neurons by reducing levels of intraneuronal Aβ and increasing levels of synaptic proteins (Tampellini et al., 2009, 2011). These outcomes are in line with the protection exerted by environmental enrichment, which has been demonstrated to enhance synaptic activity and plasticity (Eckert and Abraham, 2013), in AD mouse models (Lazarov et al., 2005; Briones et al., 2009; Gerenu et al., 2013). Activity-dependent decrease of intraneuronal Aβ might be explained with the relocation of Aβ from the inside to the outside of neurons (enhanced secretion); however, we demonstrated that degradation is also involved. The activity-dependent reduction of Aβ42, one of the most pathologic isoforms of Aβ, have been shown to occur via neprilysin (Tampellini et al., 2009), a neutral endopeptidase which is the most efficient Aβ degrading enzyme (Iwata et al., 2000). During activation, neprilysin relocates to the cell surface and shows increased colocalization with Aβ42, suggesting enhanced Aβ degradation (Tampellini et al., 2011). We are inclined to think that this pool of Aβ42 might derive from APP processing in synaptic endosomes with activation (as further discussed), and might then be transported to the neuronal surface (Rajendran et al., 2006).

During synaptic activity APP traffics anterogradly toward synapses, where it is endocytosed (Tampellini et al., 2009). This last observation complements data showing that enhanced Aβ secretion upon synaptic activation requires endocytosis (Cirrito et al., 2008). Therefore, a production of Aβ might occur at synapses with activity, as also supported by increased levels of β-C-terminal fragments (βCTFs; Kamenetz et al., 2003; Tampellini et al., 2009). Activity-dependent Aβ secretion has been observed in patients after brain injury: Aβ levels were reduced in the interstitial fluid (ISF) with worsened neurological status, and increased with improved neurological condition (Brody et al., 2008). Since, Aβ has been experimentally shown to inhibit synapses and impair synaptic plasticity (Hsieh et al., 2006; Shankar et al., 2008), one hypothesis on the physiologic role of activity-dependent Aβ secretion suggests that it might serve as feedback mechanism to prevent synaptic hyperactivation and excitotoxicity (Kamenetz et al., 2003). Intriguingly, further studies demonstrated that low concentrations (in the range of picomoles) of Aβ enhance LTP, and are involved in memory formation (Puzzo et al., 2008, 2011; Garcia-Osta and Alberini, 2009), providing evidence for a physiological function of secreted Aβ.

Altogether, the reported data suggest that, despite promoting Aβ secretion, synaptic activity might have a protective role against AD.

## Relation between synaptic activity and tau homeostasis

Tau is one of the microtubule-associated proteins that bind and stabilize neuronal microtubules during development of neuronal processes, establishment of cell polarity and intracellular transport (Binder et al., 1985; Drechsel et al., 1992; Mandelkow and Mandelkow, 1998). When phosphorylated, tau detaches from microtubules; abnormal tau phosphorylation in neurons is a hallmark of AD and other neurodegenerative diseases (including frontotemporal dementia, and progressive supranuclear palsy), and is accompanied by aggregation, and progressive intraneuronal tau accumulation. In addition to its buildup within neurons, more recent studies demonstrated that tau is also released in the extracellular space (Gómez-Ramos et al., 2006; Avila, 2010); and that increased levels of tau (total and phosphorylated) in the human's cerebrospinal fluid (CSF) are associated with an increased risk of developing AD (Blennow et al., 2010).

Tau protein is traditionally considered to be localized in axons; however, when neurons are exposed to Aβ oligomers, tau relocates to somatodendritic compartments in association with loss of spines and microtubule breakdown (Zempel et al., 2010). More recent data demonstrated the presence of tau at synapses in physiologic and pathological conditions (Pooler et al., 2014). Tau localizes in both pre and post-synaptic compartments, and the number of synaptosomes containing tau did not differ between control and AD human brains; however, a particular form of phosphorylated-tau (pS396/pS404) and tau oligomers were specifically found in AD synaptosomes (Tai et al., 2012).

Little is known on the link between tau and synaptic activity. Recent studies showed that synaptic activation enhances secretion of tau *in vitro* and *in vivo* (Pooler et al., 2013; Yamada et al., 2014). Synaptic activity was also shown to induce tau translocation to excitatory synapses, precisely in dendritic spines and post-synaptic compartments, in wild-type neurons (Frandemiche et al., 2014). In the same study, authors demonstrated that also Aβ oligomers induce tau localization to synapses; intriguingly, such translocation requires the residue S404 of tau to be phosphorylated, the same observed specifically in AD synaptosomes (Tai et al., 2012). Synaptic activation induces tau phosphorylation on residue T205; however, this phosphorylation is not mandatory for tau translocation to synapses (Frandemiche et al., 2014).

The localization of tau in dendrites is considered to be pathologic, because it is associated with loss of spines, as mentioned above (Zempel et al., 2010), and because it targets the kinase Fyn to post-synaptic compartments (Ittner et al., 2010). Fyn mediates Aβ toxicity, and its reduction or its overexpression attenuated or enhanced, respectively, synaptic alterations and cognitive impairments in AD transgenic mice (Chin et al., 2004, 2005). Fyn entry to post-synaptic compartment is tau dependent: in mice overexpressing the tau amino-terminal projection domain (PD) only (from amino acid 1 to 255, excluding the microtubule binding domain and the carboxy-terminal tail region), the tau-PD fragment does not enter dendrites, and Fyn post-synaptic targeting is diminished (Ittner et al., 2010). As result, AD transgenic mice crossed with tau-PD overexpressing mice showed decreased susceptibility to excitotoxic seizure, and improved memory (Ittner et al., 2010), suggesting protective effects when tau (and Fyn) access to dendrites is reduced. A recent paper shows that tau localization into spines is enhanced by phosphorylation. Low levels of endogenous tau are observed in dendritic spines of hippocampal neurons: when specific phosphorylation sites are replaced with glutamic acid to mimic phosphorylation (including the S404 site), tau localization in spines increases (Xia et al., 2015).

In the light of what reported so far, the translocation of tau to spines and post-synaptic compartments with synaptic activity might have negative implications for AD. However, more studies are required before ending to this conclusion. For example, what phosphorylated forms of tau are specifically present in spines and synapses during activity? Synaptic activity and Aβ seem to induce phosphorylation on different sites of tau (Frandemiche et al., 2014): perhaps, activity-induced tau phosphorylation might be more physiologic, while Aβ-induced tau phosphorylation might be more toxic. In addition, the observed activity-induced tau phosphorylation on residue T205 was reported to not be mandatory for tau targeting to synapses (Frandemiche et al., 2014), raising the possibility that tau translocation to spines with activity might occur without phosphorylation; and this would also be an intriguing subject to explore. Finally, another important question to answer is whether synaptic activation increases Fyn targeting to spines and post-synaptic compartments. Synaptic localization of Fyn has been shown to worsen the phenotype in models of β-amyloidosis; however, it has also been reported that, despite total levels of Fyn are unchanged between human AD and control, in AD brains Fyn levels are increased in somas, where it colocalizes with tau tangles, and are decreased in synaptic compartments (Ho et al., 2005), suggesting a physiologic role of Fyn at synapses.

## Functional alterations in alzheimer's disease brain: May synaptic activity be beneficial?

In the last decade several studies reported that functional alterations are common in the brain of AD patients. Even more intriguingly, neuronal dysfunction has been observed in non-demented older subjects with amyloid deposition before memory impairments (Sperling et al., 2009), which suggests it to be an early event in AD pathophysiology. Data have been provided for both increased and decreased neuronal excitability in AD patients, and in animal models of AD. Hippocampal hyperactivity has been observed in MCI patients (Bakker et al., 2012), and in young presenilin 1 (PSEN1) E280A mutation carriers (Reiman et al., 2012). Some mouse models of AD present episodes of epileptic seizure (Palop et al., 2007; Marchetti and Marie, 2011), and enhancement of GABAergic inhibitory transmission, or use of antiepileptic drugs showed protection (Sanchez et al., 2012; Verret et al., 2012; Levenga et al., 2013; Hall et al., 2015). On the other hand, reduced hippocampal activation has been observed to correlate with clinical decline in elderly (O'Brien et al., 2010). Glucose metabolism in young subject with predisposition to develop AD (ApoE4 carriers) is reduced several decades before the appearance of the first symptoms (Reiman et al., 2004), suggesting reduced brain activity. In addition, synaptic plasticity is decreased in several mouse models of AD (Trinchese et al., 2004; Shankar et al., 2008; Marchetti and Marie, 2011; Warmus et al., 2014; Menkes-Caspi et al., 2015) and by exposure to Aβ (Hu et al., 2009; Tu et al., 2014). How to interpret these mixed outcomes, and to reconcile these apparently contradictory results of reduced synaptic transmission and increased excitability in AD is still debated, as recently reviewed (Stargardt et al., 2015).

There is evidence for a protective effect of synaptic activity against AD (Swaab and Bao, 2010; Tampellini and Gouras, 2010). Higher educational attainment or participation in intellectually stimulating activities is associated with reduced risk of developing AD (Stern et al., 1994; Stern, 2006); in addition, in memory disorder clinics is common practice to encourage patients to be involved in brain stimulating activities (solving puzzles, crossed words, among others). Deep brain stimulation of the fornix in AD patients resulted in better outcomes in cognition, memory, and quality of life (Smith et al., 2012). The higher activity observed in early stages of AD might be an adaptive response boosting neuroprotection. A recent study compared brain activity of young subjects with cognitively normal older people having brain Aβ deposition: the study outcome revealed that older people had Aβ-related hyperactivation, which resulted to be a compensatory/protective mechanism reflecting neural plasticity (Elman et al., 2014).

Synaptic activity has been demonstrated to be important for neuronal survival: it is involved in the activation of survival pathways, including transcription of Activity-regulated Inhibitors of Death (AID) (a set of pro-survival genes), and resistance to apoptosis-inducing compounds (Bas-Orth and Bading, 2013). Importantly, local ATP synthesis at synapses is activity-driven, and it is fundamental for correct synaptic efficacy (Rangaraju et al., 2014). Enhanced synaptic activity was recently shown to be part of the protective mechanism exerted by rapamycin in models of AD. Rapamycin treatment increases levels of the presynaptic protein SV2 and the frequency of excitatory postsynaptic currents reducing Aβ oligomers synaptotoxicity (Ramírez et al., 2014).

The activity-dependent Aβ secretion might also work as protective mechanism to prevent Aβ buildup in the brain. One of the most accepted biomarker for AD risk/diagnosis is the decrease of Aβ42 in the CSF, which can be observed up to 10 years before conversion of MCI to AD (Buchhave et al., 2012). As observed in a study on PSEN1 mutation carriers, when Aβ42 begins to accumulate in the brain, its release in the CSF declines (Potter et al., 2013). In line with these finding, also levels of Aβ42 in the ISF are reduced in AD transgenic mouse models with aging (Cirrito et al., 2003; Hong et al., 2011). One proposed mechanism suggests that ISF Aβ42 is progressively sequestered within forming plaques in young mice; in old plaque-rich mice, ISF Aβ42 (which is less concentrated than in young mice ISF) would not derive from new biosynthesis but rather from less soluble Aβ42 deposits present in the brain parenchyma (Hong et al., 2011). Another possible mechanism to explain reduction of Aβ42 in ISF and CSF is its progressive reduced secretion by neurons. We demonstrated that AD transgenic neurons, but not wild-type neurons, secrete less Aβ1-42 in the medium, and accumulate it in distal neurites with time in culture (Tampellini et al., 2011). Thus, activity-enhanced Aβ secretion might be a physiologic event to enhance efflux of Aβ42 to the CSF. In young PSEN1 E280A mutation carriers, Aβ1-42 levels in the CSF are increased compared to control (Reiman et al., 2012). As mentioned above, the same subjects showed increased hippocampal activity; however, no differences in dementia ratings, and neuropsychological test scores were found in comparison with the control group, suggesting compensatory/protective effect of enhanced activation.

Finally, activity-induced Aβ secretion might avoid intraneuronal accumulation of Aβ, and induce new Aβ (especially Aβ42) biosynthesis, preventing mobilization of less soluble, and potentially more toxic Aβ species from the brain parenchyma.

## Conclusion

The relation between synaptic/neuronal activity and AD pathology is complex and of high interest for AD research, since it affects the homeostasis of APP, Aβ and tau, and since functional alterations can be detected very early in subjects at risk for AD.

**Figure 1:**
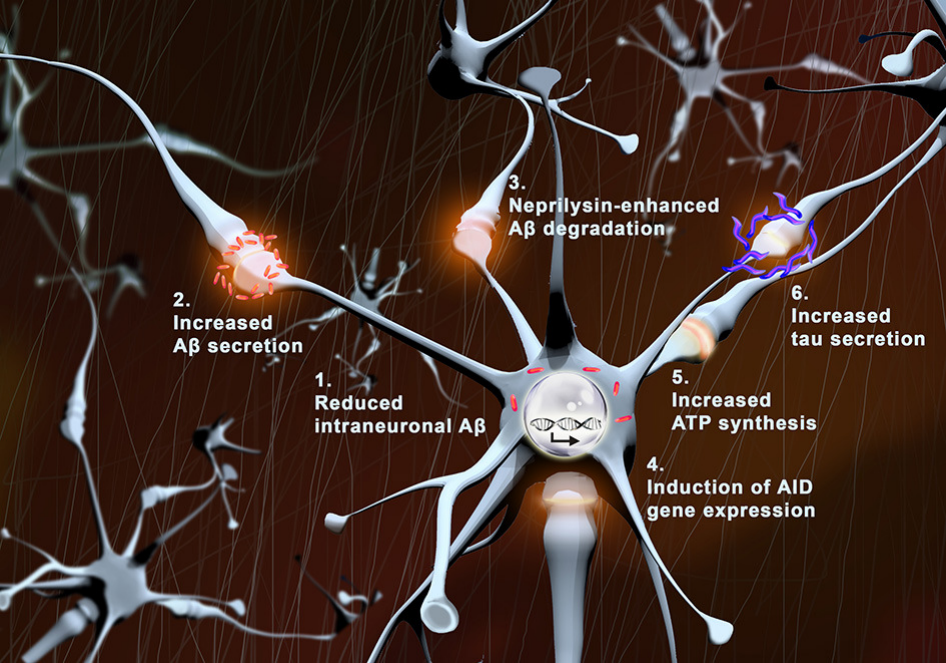
**Protective effects of synaptic activity**. With synaptic activation: (1) intraneuronal levels of Aβ (red) are reduced, (2) Aβ secretion is augmented, (3) neprilysin-induced Aβ degradation is enhanced, (4) transcription of pro-survival genes (AID) increases, (5) local ATP synthesis rises at synapses, and (6) tau secretion is augmented.

We hypothesize that physiologic synaptic activation (without induction of epileptic seizure) might be protective for neuronal preservation, and persistence of normal cognitive functions during aging (Figure 1). The higher activity observed in preclinical or early MCI patients might be the protraction of a compensatory/defensive response attempting to promote survival pathways, and preventing Aβ accumulation within neurons by maintaining its degradation and physiological secretion.

Unveiling new mechanisms linking synaptic activity with APP/Aβ and tau biology might provide new important findings on AD pathogenesis, and could lead to novel therapeutic approaches.

## Funding

The writing of this review was possible by the support of Institut Professeur Baulieu to DT.

### Conflict of interest statement

The author declares that the research was conducted in the absence of any commercial or financial relationships that could be construed as a potential conflict of interest.
